# Reducing the stress of drug administration: implications for the 3Rs

**DOI:** 10.1038/srep14288

**Published:** 2015-09-23

**Authors:** Sarah A. Stuart, Emma S.J. Robinson

**Affiliations:** 1 School of Physiology and Pharmacology, University of Bristol, University Walk, Bristol BS8 1TD, United Kingdom

## Abstract

Restraint in animals is known to cause stress but is used during almost all scientific procedures in rodents, representing a major welfare and scientific issue. Administration of substances, a key part of most scientific procedures, almost always involves physical restraint of the animal. In this study, we developed a method to inject substances to rats using a non-restrained technique. We then compared the physiological, behavioral and emotional impacts of restrained versus non-restrained injection procedures. Our results highlight the negative welfare implications associated with physical restraint and demonstrate a method which can be used to avoid this. Our work shows how adopting strategies that avoid restraint can minimize a widespread source of stress in laboratory animals and improve welfare through refinement.

The 3Rs (Reduce, Refine and Replace) are an important consideration for all scientific experiments involving animals^1^. They underpin the legislation in most countries where nationwide laws regulate the use of animals in scientific procedures as well as forming the foundation of institutional and publication guidelines^2^. Whilst major steps have been made in terms of the development of alternative methods to replace the use of animals and reduce the numbers of procedures undertaken, further improvements in animal welfare can be made through refinement of procedures^3^,^4^. One particular aspect of animal research that could benefit from refinement is the impact of restraint stress induced by substance administration techniques^5^,^6^,^7^. Stress, particularly associated which physical restraint, has been shown to cause a negative affective state in animals, as well as cardiovascular and hormonal changes^8^,^9^,^10^,^11^. Taking measures to minimise this source of stress will enhance animal welfare and could improve the variability and reproducibility of scientific data in the fields of pharmacology, toxicology and drug safety, leading to a reduction in the numbers of animals needed.

The majority of preclinical studies involve the administration of substances, and in rodents one of the most widely used routes of administration is intraperitoneal (IP). Conventional approaches for substance administration in rodents (IP, subcutaneous, gastric gavage), specified in standardized protocols, involve restraining the animal either using a firm grip around the neck and shoulder or by using a scruffing technique^12^ (Fig. 1a–c, also see http://www.jove.com/video/2771/manual-restraint-common-compound-administration-routes-mice and http://www.ahwla.org.uk/index.html; http://www.procedureswithcare.org.uk for published online guides). For rats, particularly larger animals, most guidelines recommend that the procedure is done by two people: one to restrain the animal and the other to carry out the injection^13^ (Fig. 1a) (see http://www.procedureswithcare.org.uk). Studies into the effects of IP administration and repeated experience of the technique suggest the procedure is stressful and animals show sensitization rather than habituation over repeated injections^5^,^14^. Restraint stress in animals is also a commonly used method of inducing a negative affective state and repeated restraint stress is used as a chronic stress procedure to induce a depression-like phenotype in rats^15^,^16^,^17^.

We have undertaken a study to investigate the impact of restraint on animal welfare. We hypothesized that the physical restraint used during IP substance administration is the major source of stress and aversion to the technique, rather than the injection itself. To test this hypothesis we compared effects on behavioral and physiological indicators of stress during IP injection using a conventional restraint method (Fig. 1c) and a novel method of handling involving minimal restraint to the animal (Fig. 1d, supplementary movie 1). We also utilized a new assay we developed in our laboratory to study affective states in rats, the affective bias test (ABT)^17^. This assay provides an objective measure of the effects of manipulations on an animal’s emotional state and therefore adds another dimension to our analysis of the welfare implications of restraint in rodents.

## Results

To assess stress-related behavior during IP substance administration, measurements of vocalization, escape behavior (struggling), and defecation were made by an independent observer. Since the strain^18^ and prior handling of the animal^6^,^19^ can underlie differences in stress responsiveness, we assessed male rats from different strains, ages and handling status, including well-handled Lister Hooded (n = 8), unhandled stock Wistar (n = 5), unhandled young Wistar (n = 6), unhandled ex-stud Wistar (n = 5), and unhandled young Sprague Dawley rats (n = 4). IP injections were made using saline in a 1ml/kg dose volume or, in the young, unhandled Wistar rats (n = 6), 3 mg/kg amphetamine in 1ml/kg saline, using one of the two methods. For the animals dosed with amphetamine, their behavior was scored as described above but 15 mins after injection, animals were killed and blood samples were collected and processed for corticosterone levels and plasma amphetamine.

The results from the behavioral observations revealed a main effect for struggling (Kruskal-Wallis, H_9_ = 25.5, p = 0.0025, n = 4–8 per group, Fig. 2a), vocalization (Kruskal-Wallis, H_9_ = 27.3, p = 0.0012, n = 4–8 per group, Fig. 2b) and fecal count (Kruskal-Wallis, H_9_ = 18.7, p = 0.028, n = 4–8 per group, Fig. 2c). Post-hoc pairwise comparisons also revealed a significant difference between dosing methods for the Lister-hooded rat strain for struggling scores and vocalization. As the individual group numbers were relatively small and each subgroup was fully counter-balanced, results for all animals were pooled for each method. The resulting grouped data showed that the modified dosing method was associated with a reduction in struggling (Mann-Whitney U test, U_26_ = 146.0, p < 0.0001, n = 28 per group, Fig. 2a), vocalization scores (Mann-Whitney U test, U_2_ = 119.5, p < 0.001, n = 28 per group, Fig. 2b). and fecal count (Mann-Whitney U test, U_26_ = 272.5, p = 0.01, n = 28 per group, Fig. 2c). These findings suggest that overt behaviors associated with aversion and stress are reduced in animals when handled for IP injection using our modified method. Perhaps most importantly, we also found that the modified method could be used across a range of different subgroups of animals including larger animals (>500 g) and animals that had received minimal prior handling.

In addition to the observational data, the results from the analysis of corticosterone levels showed that the modified method is associated with lower levels of this stress hormone (unpaired t-test: t_9_ = 5.74, p = 0.0003, n = 5–6 per group; Fig. 2d). Analysis of the plasma samples from the same animals also revealed no significant difference in levels of amphetamine between the groups (unpaired t-test: t_9_ = 0.43, p = 0.68, n = 5–6 per group, Fig. 2e).

In a separate cohort consisting of male Lister Hooded rats (n = 16), the rodent ABT was used to test if the restrained dosing method induced a more negative affective state relative to the modified method. These studies were all carried out by a second handler to confirm that the method was transferable between researchers. The assay was carried out as previously described^17^, with two independent learning experiences (finding a food reward in a specific digging substrate) encountered following IP dosing. One experience was learned following administration of saline (1ml/kg) using the conventional scruff method, and the other was learned following use of the modified technique. A preference test was then used to assess cognitive affective bias. The ABT has previously been used to quantify negative affective bias associated with both pharmacological and psychosocial stressors^17^. Using the ABT we showed that the conventional method of IP substance administration induces a negative affective state relative to the modified technique, resulting in an overall negative affective bias (one sample t-test: t_14_ = 2.48, p = 0.03, n = 15; Fig. 2f).

## Discussion

In this study we demonstrated and validated a new technique to minimise the use of physical restraint during administration of substances to rats by injection. These data also provide a quantitative analysis of the negative welfare implications of using restraint during substance administration and how this can be avoided. Animals handled using our modified technique were in a more positive affective state and showed lower stress hormone levels and aversive behaviors. Importantly, the bioavailability of the drug was not altered by the modified technique. Our findings show that although restraint is an accepted and often thought of as unavoidable part of undertaking procedures in rodents, it is not necessary and alternative, refined methods are achievable. In developing this refined method our studies also dissociate between the effects of discomfort from the injection and the stress associated with physical restraint.

In our study, animals showed lower levels of overt behaviours including reduced vocalization, struggling and defecation when dosed IP using the modified method. In the well-handled Lister Hooded rats almost no overt behaviours were recorded suggesting that prior habituation to handling can further enhance the benefits of this methodological adaption. These data suggest that the injection procedure itself is associated with little or no aversion (particularly in well-handled animals) and that the major stressor is the restraint used to administer the injection. It should be noted that neither method included tilting the animal so its head was lower than its abdomen as suggested in some guidance notes. Although tilting is purported to shift the viscera away from the injection site^20^, this manipulation has been called into question as the slight vacuum in the abdomen prevents the viscera from moving^20^,^21^. Tilting the animal is also likely to add to the aversive nature of the procedure and is not feasible with the modified method. Data from the analysis of plasma levels of amphetamine following either method were not significantly different suggesting that the modification to the dosing procedure did not adversely affect bioavailability.

The outcomes of the ABT provide an objective measure of the affective state of the animals and suggest that removing restraint from the drug administration procedure benefits the animal’s welfare. The negative bias towards the experience learned following injection using the restrained method suggests animals are in a more positive affective state when the modified method is used. Understanding the affective state of an animal during common laboratory procedures is a valuable way of assessing the impact of the manipulation on welfare, but until recently it has not been easily achieved^22^. Much of how we view the impact of procedures on animal welfare is based on observational analysis and some degree of anthropomorphism of what we, as humans, think is best for the animal. Within this context, the widespread use of restraint and lack of consideration of the impact this may have on experimental outcomes suggests that most researchers consider that this has minimal impact on the animal. Our results provide objective evidence that removing restraint from the method used to administer a substance is associated with animals being in a more positive affective state, and it therefore represents a major welfare advance. This work supports other studies in rats showing how alternative handling techniques, such as playful tickling, may reduce stress associated with routine injections^23^, as well as work in mice that demonstrates the beneficial effects of a more appropriate choice of handling method on animal welfare^18^.

The impact of stress on the experimental outcomes is not often discussed and yet is likely to contribute to both the primary measure in an experiment and the variability and reproducibility of the data, an area receiving much discussion in the recent literature^24^. This is particularly the case for studies involving behavioral outcomes although it is also likely to impact on other measures including *ex vivo* studies. Release of stress hormones will alter the animal’s physiological, neurochemical and psychological state as well as it’s response to drug treatments. Studies using restraint stress as an experimental manipulation, have found effects in a wide range of behavioural paradigms including drug self-administration^25^, learning and memory^26^, pain responses^27^ and food and water intake^28^. Although the period of restraint stress used during administration of substances is much shorter in duration than most experimental manipulations, the results from our corticosterone data show that even this short period of physical restraint increases stress hormones by approximately 50% above that of animals handled and injected without restraint (Fig. 2d). There are also examples of endogenous and exogenous substances exhibiting inverted U-shaped dose response relationships meaning that baseline levels of stress and arousal can dramatically alter the outcomes of the test manipulation^29^. Removing a major stress-inducing factor such as restraint could therefore benefit the experimental outcomes as well as welfare.

In the context of parenteral substance administration in rats, we have shown that refined methods which involve minimal restraint are achievable and confirm the welfare benefits that this can achieve. Our method allows for substances to be injected with minimal stress effects on the animal. The work also highlights the wider welfare implications of restraining rodents during handling and dosing procedures. Future strategies are needed to address the use of restraint within animal research and improve understanding of the implications of the associated stress on scientific data and animal welfare.

## Methods

### Animals and dosing procedures

**Figure 1:**
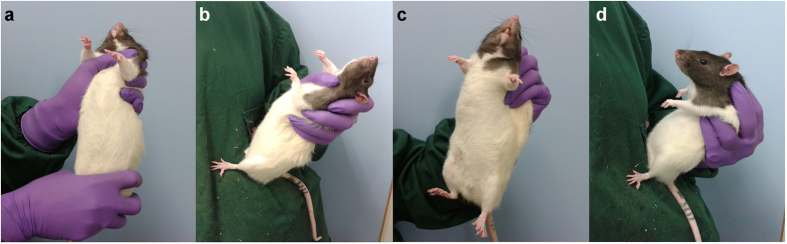
Demonstration of handling techniques for intraperitoneal dosing. (**a**) two-person restraint method, **(b)** one-person restraint, (**c**) scruff, (**d**) modified method. Also see online supplementary movie.

**Figure 2:**
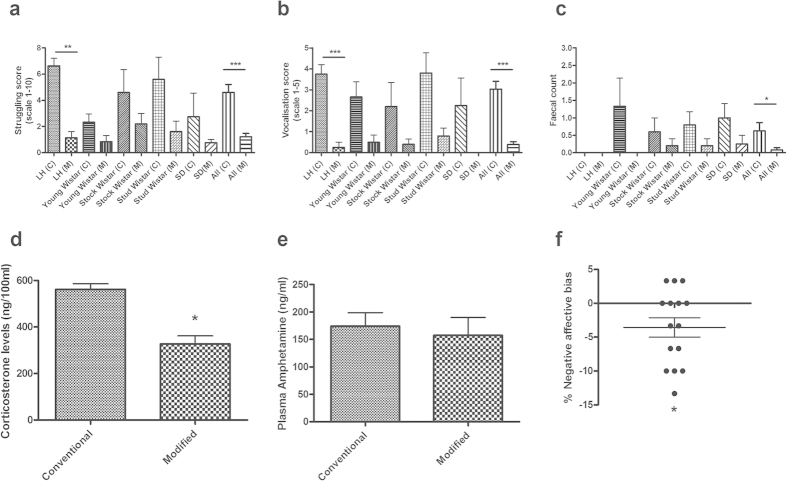
Effect of IP dosing using the conventional scruff method (C) versus the modified method (M) on behavioural, physiological and psychological measures of stress. Results for (**a**) struggling, (**b**) vocalization and (**c**) fecal counts during dosing in LH = Lister hooded, 400–550 g, n = 8 per group, young Wistar, 280-320g, n = 6 per group, Stock Wistar 400-500 g, n = 5 per group, Stud Wistar 550–700 g, n = 5 per group, and Sprague Dawley rats 290–320 g, n = 4 per group, All, n = 24 per group. Data shown as mean ± s.e.m. Plasma analysis of (**d**) corticosterone and (**e**) amphetamine for conventional (n = 6) and modified methods (n = 5; insufficient blood was collected from one animal to process). Data shown as mean ± s.e.m. (**f**) Affective bias induced by intraperitoneal dosing by the conventional versus the modified method as assessed in the ABT. Each data point represents an individual rat. Error bar, s.e.m., n = 15 rats. *p < 0.05, **p < 0.01, ***p < 0.001.

For the behavioral and physiological analysis, 5 groups of rats were tested: well-handled male Lister Hooded (Harlan, UK, 400–550 g, n = 8 per group), young male Wistar (Charles River, UK, 280–320 g, n = 6 per group), unhandled stock male Wistar (Charles River, UK, 400–500 g, n = 5 per group), unhandled stud Wistar (Charles River, UK, 550–700 g, n = 5 per group) and unhandled young Sprague Dawley rats (Charles River, UK, 290–320 g, n = 4 per group). Half the animals from each group were injected intraperitoneally (IP) using a conventional restrained method (Fig. 1c) and the other half were dosed using our modified method (Fig. 1d, also see online movie). Injections consisted of 1ml/kg saline for all groups except the young male Wistar which received 3mg/kg amphetamine (Sigma Aldrich, UK) in 1ml/kg 0.9% saline. ‘Unhandled’ refers to animals that have undergone handling for routine cage cleaning but no other habituation to human contact. ‘Well-handled’ refers to animals which had been used in behavioural studies and received regular handling including IP dosing using the modified method. Animals used for the ABT were a separate cohort of male Lister Hooded rats (Harlan, UK, 400–500 g, n = 16). All procedures were approved by the Home Office UK and conducted in accordance with the requirements of the UK Animals (Scientific Procedures) Act 1986.

### Behavioral observations

Behavioral observations of struggling, vocalizations and fecal counts were made by an independent observer. The observer scored each animal for struggling behaviour (scale 0–10, 0 = no struggling, 5 = some attempts to escape restraint during procedure, 10 = animal moving a lot and trying to escape restraint throughout procedure), vocalization (0 = no vocalization, 3 = audible vocalization for some of the procedure, 5 = audible vocalization throughout procedure) and fecal count (number of faecal pellets eliminated from start to end of the procedure).

### Plasma analysis

Animals were killed by stunning and cervical dislocation 15 min after drug administration. Immediately following cervical dislocation the animals were decapitated and the trunk blood collected into Eppendorf tubes containing 0.5M EDTA (Sigma Aldrich, UK). Samples were immediately placed on ice and then centrifuged at 4000 rpm for 10 mins to pellet cells, with plasma supernatant removed and stored at −20 °C until analysis.

Analysis of plasma corticosterone concentrations was carried out by Christian Wood and Yvonne Kershaw using radioimmunoassay. Primary rabbit anti-rat corticosterone antibody (supplied by Gábor Makara, Institute of Experimental Medicine, Budapest, Hungary) and [^125^I]-corticosterone tracer (Institute of Isotopes, Hungary) were provided by Dr Becky Conway-Campbell and Professor Stafford Lightman (School of Clinical Sciences, University of Bristol). The specific activity of the tracer was 10uCi/ml.

For the assay, a corticosterone standard curve was created using 1:2 serial dilutions of a 100ng/ml of corticosterone (Sigma-Aldrich, UK) in citrate buffer in addition to two quality controls (QCs) of 100ng/ml and 20ng/ml corticosterone. Each 10ul plasma sample was diluted to 500ul in citrate buffer (25mM tri-sodium citrate, 50mM sodium dihydrogen orthophosphate, 1g/l BSA (Sigma-Aldrich, UK)) to denature corticosterone-binding globulins. All standards, QCs and samples were processed in 100ul triplicate aliquots and were incubated at 4°C overnight containing 50ul of primary antibody (20mg antibody dissolved in 0.2ml dH_2_O, 9.8ml 0.9% saline, 500ml citrate buffer) and tracer. Following this, all samples were mixed with a dextran/charcoal T70 solution (0.05 g Dextran T70 (Pharmacia Biotech, Sweden) and 0.5 g activated charcoal (Sigma-Aldrich, UK)) and centrifuged for 15 minutes at 4000 rpm (4 °C). The supernatant for each sample was then aspirated and loaded onto a gamma counter (E5010 Cobra II Auto Gamma, Perkin Elmer, Netherlands). The assay had a limit of detection of 1ng/ml. The intra- and inter-assay coefficients of variation of the corticosterone assay were 16.7 and 13.3%, respectively.

### Plasma amphetamine analysis

Analysis of the levels of amphetamine in individual plasma samples was carried out by Ian Cummins, University of Durham. Amphetamine content was determined by gas chromatography-mass spectrometry (GC-MS) following isolation by solid-phase extraction (SPE) and derivatisation with heptafluorobutyric anhydride (HFBA), using published, validated protocols^30^,^31^ (also see Varian Certify Methods Manual, http://www.crawfordscientific.com/downloads/Application-Notes/Certify_Methods_Manual.pdf).

The method was checked for linearity, limit of detection and recovery using a supplied standard of amphetamine sulphate and control serum. Accuracy of injection was validated by injection of triplicate standards over a range of concentrations, giving an RSD of 2%. Single ion monitoring (SIM) of characteristic fragment ions was used to quantify amphetamine and the SIM data for m/z 118 and m/z 240 were combined.

### Affective bias test training and testing procedure

The animals were trained and tested in a Perspex arena, 40 cm^2^. The substrates e.g. bedding, sawdust, sand, cloth, perlite etc., were placed in glazed pottery bowls (5 inch) and presented in a pseudo-random spatial order to prevent rats using spatial cues to select the correct substrate.

#### Training

The rats were habituated to the test arena and trained to dig in two bowls filled with sawdust to obtain a quantity of food pellets (45mg rodent tablet, TestDiet). Training was complete once each rat was able to find the pellet in each bowl on 12 consecutive trials within 5 minutes.

#### General ABT Protocol

The study followed a standard protocol of four pairing sessions followed by a preference test session on the fifth day. Rats were randomly assigned to one of four counterbalanced groups to prevent bias associated with substrate, treatment, or treatment day. Each pairing session consisted of individual trials in which the rat was required to choose between one of the two bowls to locate a food pellet reward. In each of these trials, one of the bowls contained a ‘reward-paired’ substrate (A) and the other contained a different, ‘blank’ substrate (C). The blank substrate was the same for all four pairing sessions. In the blank substrate, the equivalent number of food pellets was crushed into the bowl to avoid discrimination from the reward-paired substrate based on odour. The rat was placed in front of the two bowls and allowed to dig in one of the two bowls. Once the animal began to dig, the other bowl was removed from the test arena. Digging in the reward-paired substrate was recorded as a correct trial, and digging in the blank substrate was recorded as an incorrect trial. The latency to dig was also recorded for each trial and the session was completed once the rat reached a criterion of 6 consecutive correct trials (probability of by chance being 0.015). The second pairing session followed the same protocol, but the rats were presented with the second reward-paired substrate (B). The pairing sessions were repeated to give a total of four sessions on consecutive days. On the fifth day, the rats were presented with both reward-paired substrates for a total of 30 trials. A single pellet reward was placed in one of the bowls using a random reinforcement protocol such that there was a 1 in 3 reward probability for each substrate. The random reinforcement schedule was used to maintain animals responding during the preference test but reduce the potential confounds associated with new learning.

#### Restraint-induced stress

For each rat, one substrate (A or B) was paired immediately following dosing with saline using the conventional scruff method (Fig. 1c), and the other was paired following use of the modified technique (Fig. 1d).

#### Statistical Analysis

All data were analysed using GraphPad Prism (Version 5). Results from the behavioural observations were analysed using a one way ANOVA, non-parametric Kruskal-Wallis test. Planned pairwise comparisons for each sub-group and method were made using a Mann-Whitney *U* tests. In addition, data for each method was pooled for the different sub-groups tested and the resulting two groups compared using a two-tailed Mann-Whitney *U* tests. Results from the plasma analysis of serum corticosterone and amphetamine levels were analysed using a two-tailed, unpaired t-test. Data from the affective bias test were recorded as the % negative bias, which was calculated from the number of choices made for the substrate paired during restrained dosing versus the number of choices made for the substrate paired during the modified method. The resulting data were analysed using a one sample t-test against the theoretical mean of 0% choice bias, equivalent to no preference for either substrate.

## Additional Information

**How to cite this article**: Stuart, S. and Robinson, E. S.J. Reducing the stress of drug administration: implications for the 3Rs. *Sci. Rep.* **5**, 14288; doi: 10.1038/srep14288 (2015).

## Supplementary Material

Supplementary Movie

Supplementary Movie 1
