# Corrigendum: Reducing the stress of drug administration: implications for the 3Rs

**DOI:** 10.1038/srep17116

**Published:** 2015-12-14

**Authors:** Sarah A. Stuart, Emma S.J. Robinson

Scientific Reports 5: Article number: 1428810.1038/srep14288; published online: 09 23 2015; updated: 12 14 2015

Review-only material was inadvertently published with the original version of this Article as Supplementary Movie 1. This has now been removed.

